# Thermoelectric Power-Factor of Ag-Doped TiO_2_ Thin Film

**DOI:** 10.3390/mi13122169

**Published:** 2022-12-08

**Authors:** Rohaida Usop, Megat Muhammad Ikhsan Megat Hasnan, Mahazani Mohamad, Mohd Khairul Ahmad, Suhana Mohd Said, Faiz Salleh

**Affiliations:** 1 Faculty of Engineering, Universiti Malaya, Kuala Lumpur 50603, Malaysia; 2 Faculty of Engineering, Universiti Malaysia Sabah, Kota Kinabalu 88400, Malaysia; 3 Faculty of Electrical and Electronic Engineering, Universiti Tun Hussein Onn Malaysia, Parit Raja 86400, Malaysia

**Keywords:** titanium dioxide, silver, rutile phase, nanostructure, doping, thermoelectric power-factor, thermoelectric

## Abstract

The thermoelectric power-factor of two types of rutile-phased nanostructured-TiO_2_ thin films doped with Ag was investigated at room temperature, by measuring their Seebeck coefficient and electrical conductivity. The thin films, consisting of a nanorod structure (single layer) and nanorod and nanoflower structure (bilayer) of TiO_2,_ with the addition of different wt.% of AgNO_3_ were synthesized on an F:SnO_2_-coated glass substrate. The evaluated thermoelectric power-factor was observed to increase with an increasing wt.% of AgNO_3_ for both structures, with the bilayer structure increasing three times more than the undoped bilayer-structure, with a value of 148 μWm–^1^K^–2^ at 0.15 wt.%. This enhancement was due to the increase in electrical conductivity, which compensated for the small changes in the Seebeck coefficient, which were likely due to the increase in carrier concentration. Consequently, an enhancement in the thermoelectric conversion-efficiency of TiO_2_ thin film may be observed by Ag doping, without influencing the layer structure and material phase.

## 1. Introduction

TiO_2_ material is considered a good candidate for niche thermoelectric applications that require its good transparency, controllable wettability, excellent ultraviolet-protective ability, and non-toxicity, despite its low thermoelectric conversion-efficiency compared with the conventional thermoelectric material [1,2,3]. It is theoretically reported that rutile-phased TiO_2_ has the highest figure-of-merit *Z*, which determines the thermoelectric conversion-efficiency compared with its other polymorphs [4]. The *Z* is defined as the ratio of the thermoelectric power-factor (PF, product of the Seebeck coefficient *S*, squared, and electrical conductivity *σ*) to the thermal conductivity *κ*. The *Z* is also known to be strongly dependent on the carrier concentration, with the theoretical optimal *Z* of rutile-phased TiO_2_ being at a carrier concentration of ~10^21^ cm^−3^ [4,5]. Thus, the alteration of carrier concentration is one of the approaches to enhance the thermoelectric conversion-efficiency of TiO_2_. The enhancement is also experimentally reported by increasing the carrier concentration through oxygen deficiency and doping, which increase *σ*, and by promoting scattering processes such as grain-boundary and point-defect scatterings, which reduce the *κ* [6,7,8,9,10]. Okinaka et al. have reported that the dimensionless figure-of-merit *ZT* of defect-controlled TiO_X_ is 1.64 at 1073 K, which is among the largest *ZT* values reported in this temperature range compared with other oxide materials such as co-doped ZnO of 0.65 at 1247 K, W-doped MnO_2_ of 0.25 at 1223 K, and Ca_3_Co_4_O_9_ of 0.87 at 973 K [11,12,13,14,15]. These show that TiO_2_ material has a huge potential for application as thermoelectric material.

Moreover, the enhancement in *Z* is also expected by introducing the nanostructure, in which the introduction of the nanostructure is able to simultaneously increase *S* and reduce *κ* [16]. The introduction of ordered mesoporous TiO_2_ is reported to reduce *κ* significantly by inducing phonon scattering [17]. However, the mesoporous structure is reported to be affected by doping the ordered mesoporous TiO_2_ with metal impurities, in order to improve its *σ* [17]. Thus, it is necessary to preserve the nanostructure while attempting to increase the *σ* by doping, to preserve both benefits. Furthermore, the introduction of co-doping was also reported to improve the PF of TiO_2_ nanostructured-bulk material, in spite of the small increase in *κ*, and the introduction of nanoparticles on TiO_2_ nanotubes was also reported to improve the *S* [18,19]. Therefore, it is important to preserve the nanostructure of TiO_2_ for reducing the *κ*, while improving its PF by doping. In our previous study, we measured *S* and *σ* of two types of TiO_2_ thin films; a single layer consisting of nanorod TiO_2_ and a bilayer consisting of nanorod and nanoflower TiO_2_ [20]. It was found that the measured *S* and *σ* of TiO_2_ thin films depend on the type of layer structure and are independent of the layer’s thickness. Moreover, the PF of nanostructured-TiO_2_ thin film was also found to be lower than that of bulk TiO_2_. Therefore, in the present paper, in order to enhance the PF of a nanostructured-TiO_2_ thin film by increasing the carrier concentration through doping while maintaining the layer’s nanostructure, two types of rutile-phased nanostructured-TiO_2_ thin films, with the addition of different weight percentages, wt.%, of AgNO_3,_ were synthesized on F:SnO_2_-coated (FTO) glass substrate. The *S* and *σ* of the synthesized samples were measured, in order to clarify the influence of doping on the PF.

## 2. Experiment

The TiO_2_ thin films discussed in this paper were synthesized using the typical hydrothermal method, and all chemicals were used without any purification [20]. A mixed solution of 120 ml of deionized water and 120 ml of HCl (36.5–38%, J. T. BAKER, Phillipsburg, NJ, USA) was prepared and stirred for 5 min. AgNO_3_ with different wt.% was added to the solution and stirred for 30 min at a temperature of 220 °C. A total of 5 ml of C_16_H_36_O_4_Ti (TBOT, Sigma-Aldrich, St. Louis, MO, USA) was added dropwise to the solution after the stirred solution was cooled to room temperature and the solution was then stirred until it became homogenous. The solution was then inserted into a Teflon-lined stainless-steel autoclave (Nas Sutera Engineering, Johor, Malaysia), which consists of an FTO glass substrate that is placed inside it. The Teflon-lined stainless-steel autoclave was placed in an oven at 150 °C for 10 hours. After it was cooled to room temperature, the synthesized sample was washed several times with deionized water and dried in an oven at 70 °C for 30 min. In this paper, the two types of synthesized TiO_2_ thin-film structures will be referred to as the NR and the NRFs sample, for the single layer consisting of a nanometer-sized rod TiO_2_ and the bilayer consisting of a nanometer-sized rod and flower of TiO_2_, respectively.

The field-emission scanning electron microscopy (FESEM, JEOL JSM-7600F, JEOL Ltd., Tokyo, Japan) equipped with energy-dispersive X-ray (EDX) spectroscopy was used at an accelerating voltage of 5.0 kV, for investigating the surface methodology and determining the elemental composition of the synthesized samples. The X-ray diffractometer (XRD, PANALYTICAL X’PERT3 POWDER, Malvern Panalytical, Worcestershire, UK) was used to identify the phase and composition of the samples by collecting their XRD patterns at a scan rate of 2°/min over the range of 20° to 50°, with Cu Kα radiation (λ = 1.5418 Å) at a glancing angle of 5°. The conventional S and electrical-resistance measurement system (ULVAC, ZEM-3, ADVANCE RIKO Inc., Yokohama, Japan) was used for measuring the thermoelectromotive force, temperatures, and electrical resistivity of the samples near room temperature. The *S* of the samples was evaluated from the gradient of the linear relation between the simultaneously measured thermoelectromotive-force and temperature difference. By considering the thickness and the surface area of the cross-section of the TiO_2_ layer estimated from the FESEM image, the *σ* was evaluated from the measured electrical-resistance. The PF was calculated using the evaluated *S* and *σ* near room temperature. The Hall measurement was carried out at room temperature by using the Hall-effect measurement system (DEXING MAGNET DX-100, Xiamen Dexing Magnet Tech. Co. Ltd., Xiamen, China) under a magnetic field of 0.5 T, for determining the carrier concentration of the samples. The band-gap energy of the samples was determined, using the Kubelka–Munk theory, from the diffuse-reflectance spectra measured by using ultraviolet–visible-near-infrared (UV-VIS-NIR) spectroscopy (AVANTES, Apeldoorn, Netherlands) at room temperature and in the range of wavelengths of 290–490 nm [21]. The transmission spectra of the samples were analyzed by using ultraviolet–visible-near-infrared (UV-VIS-NIR) spectroscopy (AVANTES) in the range of wavelengths of 370–700 nm.

## 3. Results and Discussion

**Figure 1:**
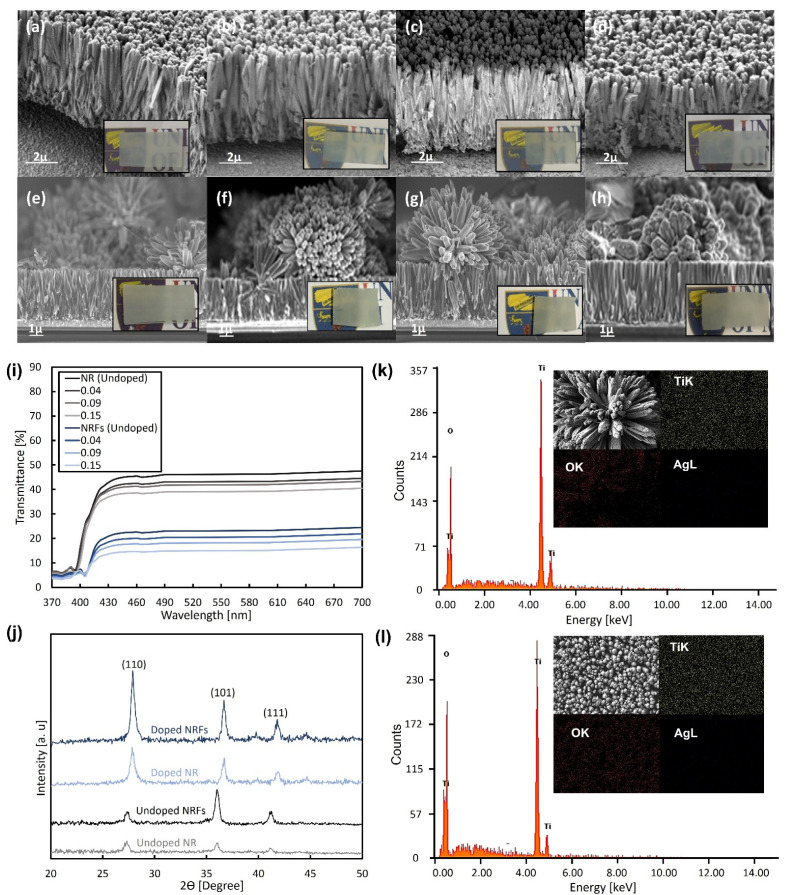
FESEM images of NR samples for (**a**) 0, (**b**) 0.04, (**c**) 0.09, and (**d**) 0.15 wt.% of AgNO_3_, and NRFs samples for (**e**) 0, (**f**) 0.04, (**g**) 0.09 and (**h**) 0.15 wt.% of AgNO_3_. The insets show photographs of each sample. (**i**) Transmission spectra of the synthesized NR and NRFs samples with additions of 0, 0.04, 0.09, and 0.15 wt.% of AgNO_3_. (**j**) XRD patterns of the synthesized NR and NRFs samples for 0 (undoped) and 0.15 (doped) wt.% of AgNO_3_. EDX spectrum of (**k**) NRFs and (**l**) NR samples with 0.15 wt.% of AgNO_3_. The insets show FESEM images and elemental mapping of Ti, O and Ag for each sample. (NR sample: single layer consisting of nanometer-sized rod TiO_2_ and NRFs sample: bilayer consisting of nanometer-sized rod and flower of TiO_2_).

The FESEM images of synthesized NR and NRFs samples with different wt.% of AgNO_3_ are shown in Figure 1, and the insets show the photographs of each sample. From Figure 1a–h, it is observed that the NR and NRFs samples are composed of a dense nanometer-sized rod-like structure and a rod- and flower-like structure, respectively. The formation of NR and NRFs structures is controlled by changing the direction of the FTO-conducting surface when placing it inside the Teflon-lined stainless-steel autoclave [20]. The direction is considered to promote the growth of nanorod TiO_2_ in the direction normal to the substrate surface or the settlement of nanoflower TiO_2_ on the nanorod-TiO_2_ layer. The thicknesses of the NR and NRFs layers are also observed to be approximately ~4 and ~8 µm, respectively. Figure 1i shows the transmittance of undoped NR and NRFs samples, together with the doped samples of 0.04, 0.09, and 0.15 wt.% of AgNO_3_ in the 370–700 nm wavelength range. It is observed that the transmittance of the NR and NRFs samples is ~40% and ~18%, respectively, in the region above ~420 nm. An increase in the doping concentration leads to a small decrease in the sample’s transmittance for both the NR and NRFs samples. The NR samples showed larger transmittance in the visible-wavelength range than the NRFs samples, which is consistent with the photographic images in Figure 1a–h. Thus, it is considered that the structure of these uniformly deposited NR and NRFs layers on FTO-glass substrates and their transmittance are not significantly affected by the addition of a small amount of AgNO_3_.

Figure 1j shows the XRD patterns of NR and NRFs samples with 0 (undoped) and 0.15 (doped) wt.% of AgNO_3_. It is found that the XRD patterns for undoped NR- and NRFs-samples indicate rutile-phase TiO_2,_ by exhibiting the diffraction peaks at 27.31°, 36.02° and 41.07°, which correspond to the (110), (101) and (111) planes, respectively [22]. However, the diffraction peaks for the doped NR and NRFs samples with 0.15 wt.% of AgNO_3_ are shifted to a higher angle. These shifts of the diffraction peaks are consistent with reported Ag-doped TiO_2_ cases, which are considered to be due to the changes in the lattice parameter resulting from the incorporation of different ionic radii of metal ions into the crystal lattice [23,24]. Moreover, the diffraction peaks of Ag at 38.2° and 44.5° are also hardly observed, which suggests that Ag is homogeneously doped and dissolved into the TiO_2_ rutile-structure [24]. From Figure 1j, it is also observed that the intensity of the peak increases with the introduction of Ag doping, which is expected to occur at lower dopant concentrations, since Ag is considered to improve the rutile phase, and will start to degrade the rutile phase when the concentration is higher than 1.0 wt.% [23,25]. For the NRFs sample, a significant increase in the peak which corresponds to the (110) plane is observed, while a small increase in peak is observed for the (101) plane. This is likely due to the reduction of deposited nanoflowers. However, the reduction is hardly seen in the FESEM image shown in Figure 1h, which needs further investigation. Figure 1k,l show the EDX-spectrum analysis for the NR and NRFs samples with 0.15 wt.% of AgNO_3_ at 15 keV of electron-beam energy. The insets show the corresponding top FESEM-image and the elemental mapping of the Ti, O and Ag elements. From Figure 1k,l, it is observed that the peak of the Ag element at 3 keV is not detected, which is likely due to the very low wt.% of AgNO_3_ used in the synthesis process [23]. From the insets of Figure 1k,l, the distribution of the Ag element is also hardly seen in both samples, in contrast to the Ti and O elements, which are observed to be uniformly distributed in both samples. Thus, it is considered that the Ag is doped in the NR and NRFs layer, without significantly affecting the phase and composition of rutile-phased TiO_2_.

**Figure 2:**
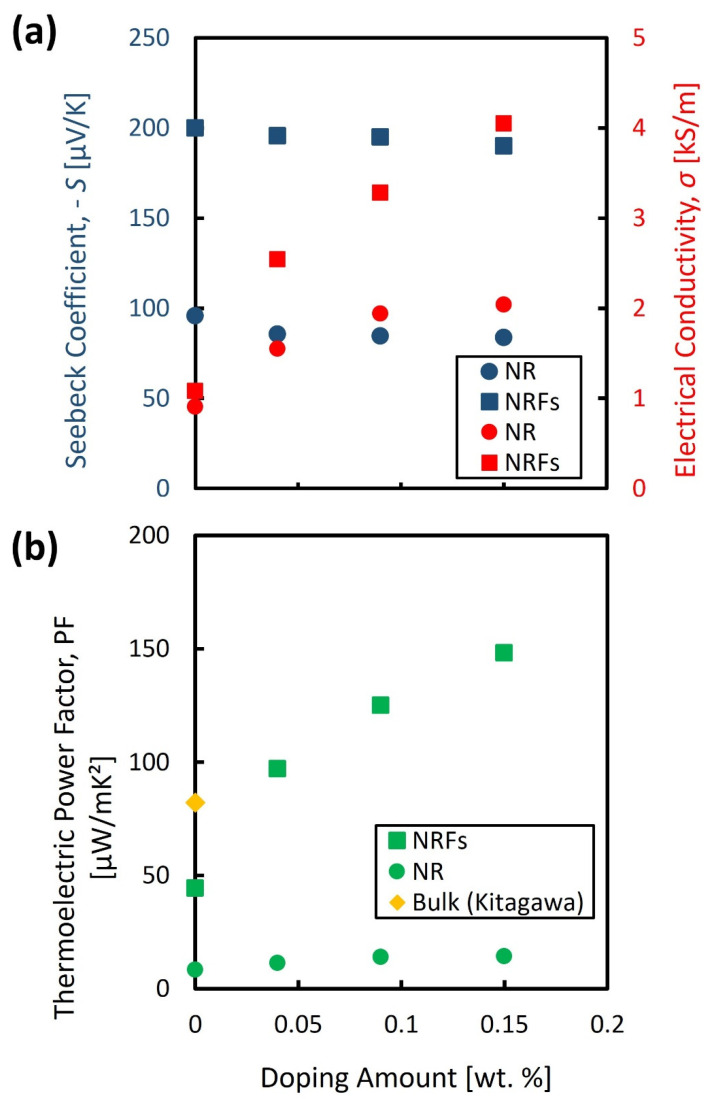
Evaluated (**a**) *S* and *σ*, and (**b**) PF, as a function of wt.% of AgNO_3_. The filled circle ● and square ■ represent the evaluated value for NR and NRFs samples, respectively. The filled blue and red points represent the *S* and *σ*, respectively. The filled diamond ♦ represents the reported value for bulk TiO_2_ which is adapted from [9].

The evaluated *S* and *σ* are shown in Figure 2a as a function of wt.% of AgNO_3_. The filled circle and square represent the evaluated values for the NR and NRFs samples, respectively. The filled blue and red points represent the S and σ, respectively. From Figure 2a, the polarity of *S* for both samples is negative, and the polarity is observed to be maintained by increasing the wt.% of AgNO_3_. These results indicate that the NR and NRFs samples are n-type semiconductors, which is also further confirmed by the Hall measurement. The majority carriers resulted in the negative polarity of the *S* of both samples, and are considered to originate from the oxygen vacancy [26]. Moreover, the *S* of both samples is found to be slightly decreased with the increase in wt.% of AgNO_3_. In contrast with that slight reduction in *S*, the *σ* of both samples is found to increase with the increase in wt.% of AgNO_3_. This interdependence of *S* and *σ*, and their dependency on impurity concentration are in good agreement with that usually observed in semiconductor material.

**Figure 3:**
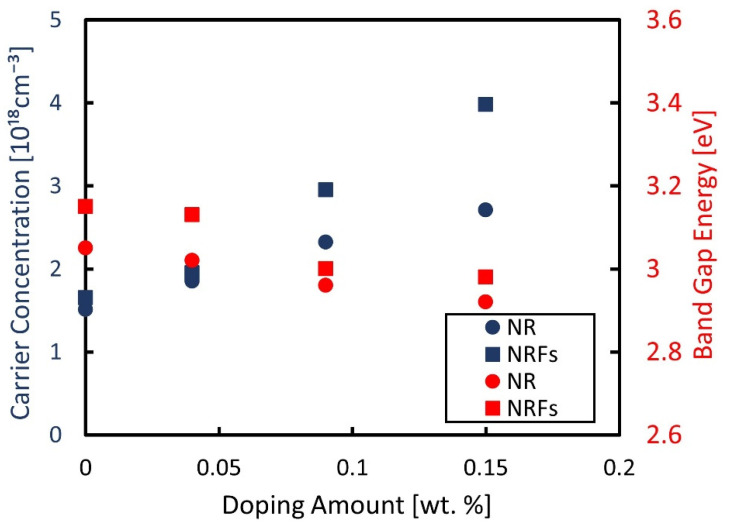
Measured carrier-concentration and evaluated band-gap energy as a function of wt.% of AgNO_3_. The filled blue and red points represent the measured carrier-concentration and the band-gap energy, respectively. The filled circle ● and square ■ represent the NR and NRFs samples, respectively.

**Figure 4:**
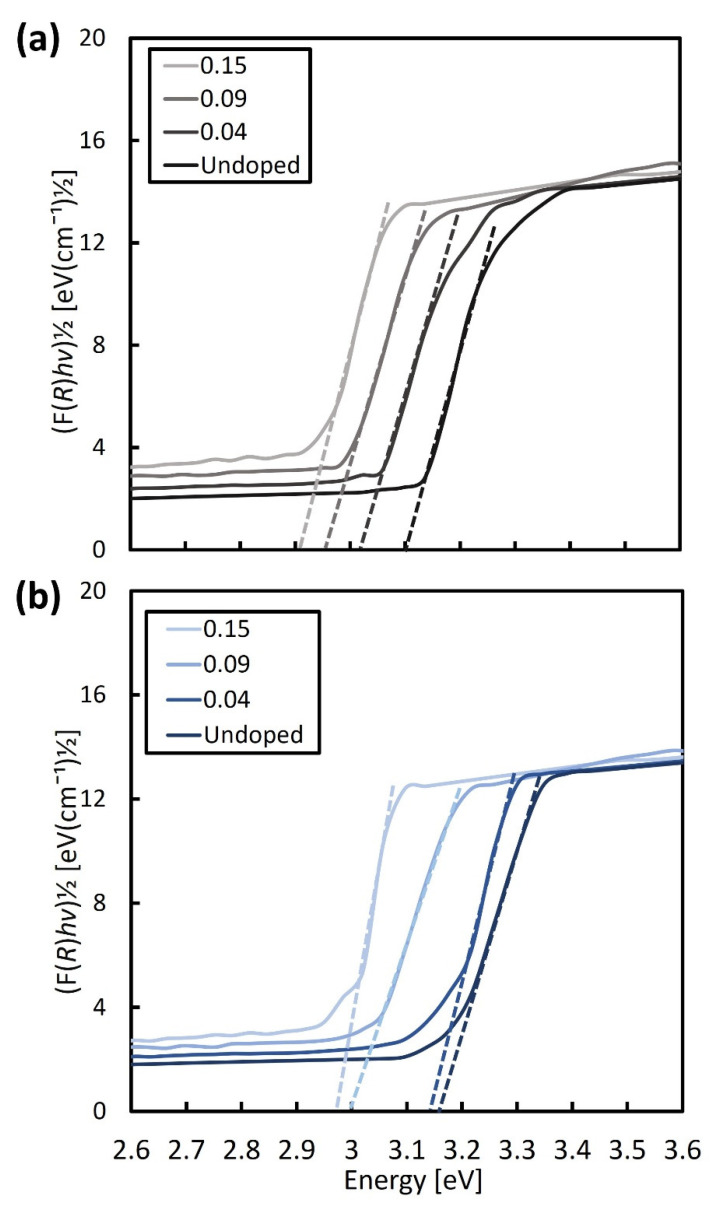
Kubelka–Munk function as a function of photon energy for (**a**) NR and (**b**) NRFs samples with additions of 0 (undoped), 0.04, 0.09, and 0.15 wt.% of AgNO_3_. The broken lines were shown to intercept the phonon-energy axis for assessing the band-gap energy of NR and NRFs samples.

Figure 3 shows the measured carrier-concentration of the samples, using a Hall-effect measurement system, and the evaluated band-gap energy considering the Kubelka–Munk theory as a function of wt.% of AgNO_3_. The band-gap energy was determined from the plotted Kubelka–Munk function (F(*R*)*hν*)^1/2^ as a function of the photon energy, *hν*, shown in Figure 4a,b for the NR and NRFs samples, respectively. From Figure 3, it is found that the carrier concentration of both samples increases with an increase in the wt.% of AgNO_3_, with the NRFs sample showing a higher increment, compared with the NR sample. It is reported that the introduction of Ag in TiO_2_ increases the lower reduction-states of Ti, which promote the oxygen vacancy formed from the surface defects [23,27,28]. This statement is in agreement with the reduction of evaluated band-gap energy shown in Figure 3 for both samples, which is considered to be due to the synergistic effect between the Ag dopant and vacancy defect [23]. That is, it is considered that the decrease (increase) in *S* (*σ*) with an increase in the wt.% of AgNO_3_ is likely due to the increase in carrier concentration.

Figure 2b shows the calculated PF as a function of wt.% of AgNO_3_. The filled circle and square represent the evaluated values for the NR and NRFs samples, respectively. The filled diamond represents the reported value for bulk TiO_2_ [9]. From this figure, it is found that the PF for both samples increases with an increase in the wt.% of AgNO_3,_ as reflected by the increase in *σ*, which compensates for the slightly decreased value of *S*. The PF of the NRFs (NR) sample with the addition of AgNO_3_ as low as 0.15 wt.% is three (or two) times larger compared with the undoped NRFs (NR) sample, and almost two times larger compared with the reported bulk TiO_2_. From Figure 2b, it is considered that the high increase in PF of the doped NRFs sample is mainly governed by the high increase in *σ*. One of the possible reasons for the higher increase of *σ* in the NRFs sample is the structure of the NRFs sample, which likely promotes more surface defects compared to the NR structure. It should also be noted that a significant reduction in κ is also expected by introducing the nanometer-sized structures in the TiO_2_ film. Therefore, the improvement of PF in NR and NRFs samples by Ag doping without affecting their nanometer- sized structure (preserving the expected reduction in *κ*), might be one of the approaches for further enhancing the thermoelectric conversion-efficiency of the TiO_2_ material for niche thermoelectric-application.

## 4. Conclusions

In order to enhance the PF of a nanostructured-TiO_2_ thin film by increasing the carrier concentration through doping while maintaining the layer’s nanostructure, we synthesized two types of rutile-phased nanostructured-TiO_2_ thin films with the addition of different wt.% of AgNO_3_ on an FTO-glass substrate. The two types of synthesized-TiO_2_ thin films have the structure of a single layer and a bilayer, with dense nanometer-sized rod-like structures and rod- and flower-like structures, respectively. The measured *σ* (*S*) of both samples is found to increase (decrease) with an increase in the wt.% of AgNO_3,_ which is in good agreement with that usually observed in semiconductor material. Moreover, the NRFs sample is found to possess the highest PF with an addition of AgNO_3_ as low as 0.15 wt.%, which is three times larger compared with the undoped NRFs sample, and almost two times larger compared with the reported bulk TiO_2_. These enhancements in PF are likely due to the promotion of oxygen vacancies formed from the surface defects, which increase the *σ*. Consequently, the enhancement of PF in nanostructured-TiO_2_ thin film by Ag doping without significantly affecting its nanostructure has been demonstrated, which will provide a promising TiO_2_ thermoelectric-material for niche thermoelectric applications.
